# Post-Moore Memory Technology: Sneak Path Current (SPC) Phenomena on RRAM Crossbar Array and Solutions

**DOI:** 10.3390/mi12010050

**Published:** 2021-01-03

**Authors:** Ying-Chen Chen, Chao-Cheng Lin, Yao-Feng Chang

**Affiliations:** 1 School of Informatics, Computing and Cyber Systems, Northern Arizona University, Flagstaff, AZ 86011, USA; 2 Taiwan Semiconductor Research Institute, National Applied Research Laboratories, Hsinchu 30078, Taiwan; chcelin@narlabs.org.tw; 3 Department of Electrical and Computing Engineering, The University of Texas at Austin, Austin, TX 78712, USA; yfchang@utexas.edu

**Keywords:** selectorless, resistive switching, sneak path current, volatile, resistive random access memory (RRAM)

## Abstract

The sneak path current (SPC) is the inevitable issue in crossbar memory array while implementing high-density storage configuration. The crosstalks are attracting much attention, and the read accuracy in the crossbar architecture is deteriorated by the SPC. In this work, the sneak path current problem is observed and investigated by the electrical experimental measurements in the crossbar array structure with the half-read scheme. The read margin of the selected cell is improved by the bilayer stacked structure, and the sneak path current is reduced ~20% in the bilayer structure. The voltage-read stress-induced read margin degradation has also been investigated, and less voltage stress degradation is showed in bilayer structure due to the intrinsic nonlinearity. The oxide-based bilayer stacked resistive random access memory (RRAM) is presented to offer immunity toward sneak path currents in high-density memory integrations when implementing the future high-density storage and in-memory computing applications.

## 1. Introduction

With the high demand for high-density memory storage applications, alternative memory technology has been intensively investigated for replacing conventional charge-based flash memory which suffers from charge loss and program errors with device scaling down. To break the bottle-neck of device scaling, several emerging memory technology attracts considerable attention, such as phase-change memory (PCM) [1], ferroelectric random access memory (FeRAM) [2,3], magnetic resistive random access memory (MRAM) [4], and resistive random access memory (RRAM). Among them, the resistive random access memory (RRAM) device holds great potential as an emerging candidate because of its simple design, high-speed operation, excellent scalability, and low power consumption [5,6,7,8]. A resistive random access memory cell, namely memristor, is a nonlinear, passive two-terminal resistance component associated with the combination of charge and magnetic flux, proposed by Leon Chua in 1971 and has been deepened into practical applications by HP Labs [9].

The metal-insulator-metal (MIM) structure exhibits resistance switching (RS) characteristics which result from the inevitable existence of non-stoichiometry in insulating metal oxide thin film. Among the metal oxide insulators, hafnium oxide (HfO_x_) was proposed as the most promising material system based on the updated International Technology Roadmap for Semiconductors (ITRS) due to its overall performances on reliability including endurance property (>10^12^ cycles), retention (>10 years at 85 °C), and operation stability (i.e. current limiting operation) conformed with the requirements of non-volatile memory and storage-class memory applications [10]. Besides, HfO_x_ has been extensively studied and used as the gate dielectrics for MOSFETs since 45 nm and 22 nm technology node and is compatible with the complementary metal-oxide-semiconductor (CMOS) process.

In the era of computational and information technologies renovation, such as artificial intelligence (AI), internet-of-things (IoT), edge computing, in-memory computing paradigm, etc., the emerging memory technology with high-density storage, large bandwidth, and low power consumption has also been rapidly explored over years. With the simple crossbar structure featuring 4F^2^ of footprint, RRAM is also a promising candidate for next-generation nonvolatile memory technologies for both data storage and in-memory computing owing to its ultra-low power consumption, simple structure, and high scalability for 3D integration of the computing and memory elements, e.g. 3D vertical RRAM [11,12,13,14]. Speaking of device architecture, RRAM has an advantage for 3D cross-point architecture beyond planar (2D) structure to maximize memory density in the minimum active area [15,16]. However, sneak-path current (SPC) is a general inevitable problem in crossbar RRAM configuration, and significantly affect the read operation because each word and bit lines are connected in perpendicular directions. That is, the interference currents from neighboring cells result in reading error and false programming. Consequently, to access a selected memory cell in the cross-point array with interference robustness and SPC immunity is the major challenge for high-density memory arrays [17,18]. Consequently, reading a selected cell within the cross-point array without interference from leakage current through neighbor cells is the major roadblock to accomplishment the high-density memory cell arrays. 

To address the issues results from SPC, various selection devices has been presented for the one-transistor-one-on-memory (1T1R) (6F^2^) or one-selector-one-memory (1S1R) configuration [19,20,21,22], where each memory component is companied with a switch devices such as conventional transistor or diode, oxide-based metal-insulator-transition switch [23,24,25], volatile switching selector [21], chalcogenide-based threshold switching devices [26,27,28], mixed-ionic-electronic-conduction (MIEC) selector [29] etc. Despite the 1T1R configuration is considered a solution for eliminating the sneak path currents, it compromises the advantage of ultrahigh scalability of RRAM in the cost-manufacture effective approach for high-density storage applications. Therefore, it is of great interest to investigate the sneak path current in a crossbar array, and develop solutions includes not only selection device for integration but a self-rectifying memory for high-density storage application. 

In this paper, the sneak path current is observed and investigated with operation modulations on varied RRAM memory structure designs. The methodology of conducting the electroporation by voltage sweeping on the RRAM crossbar array has been presented as followed by the read interference and cross-talk observations. The self-rectifying resistive switching behavior in the bilayer stacked devices through designed operation conditions is presented with SPC immunity, which can be leveraged and become the solution to realize three-dimensional memory configuration for high-density memory storage applications and high-demanding computational architectures. 

## 2. Materials and Methods 

**Figure 1:**
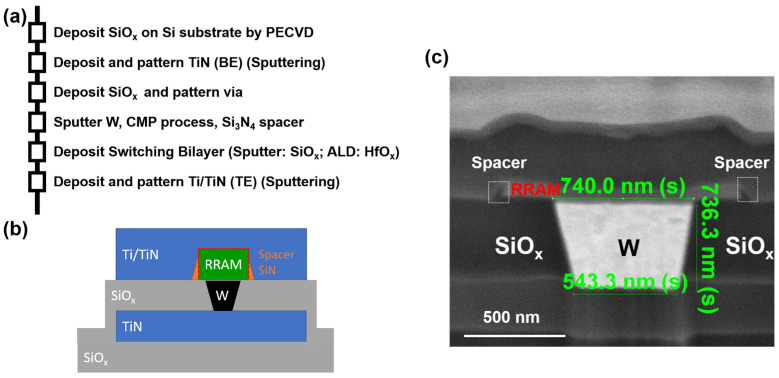
(**a**) Fabrication process flow for the two-terminal memory with tungsten plug as a bottom electrode (BE) and TiN as a top electrode (TE), (**b**) schematic of two-terminal memory with TiN spacers, (**c**) TEM image of the two-terminal memory.

Figure 1 shows the fabrication process flow, schematic of the device structure, and transmission electron microscopy (TEM) image (cross-section) of the RRAM device, respectively. The heavily-doped N+ Si wafers are as the starting substrates with SiO_x_ deposition by plasma-enhanced chemical vapor deposition (PECVD), titanium nitride (TiN)) stack of 200 nm deposited as the bottom electrode. The SiO_x_ isolation layers deposited by PECVD, and via was patterned by photolithography. The via is composed of PECVD deposited SiO_x_ of ~740 nm. The via sizes in square length are 400 nm, 750 nm, and 1 µm. The tungsten (W) was filled and deposited by the radio frequency (RF) sputtering method, followed by the chemical mechanical polishing (CMP) process. Due to the higher material reaction rate toward the slurry of the CMP process, the tungsten has been polished more than SiO_x_ isolation layers surround the via. The silicon nitride (SiN) was deposited and patterned as the spacers for improving electrical isolation between the top and bottom electrodes and increasing the device yield. 

**Figure 2:**
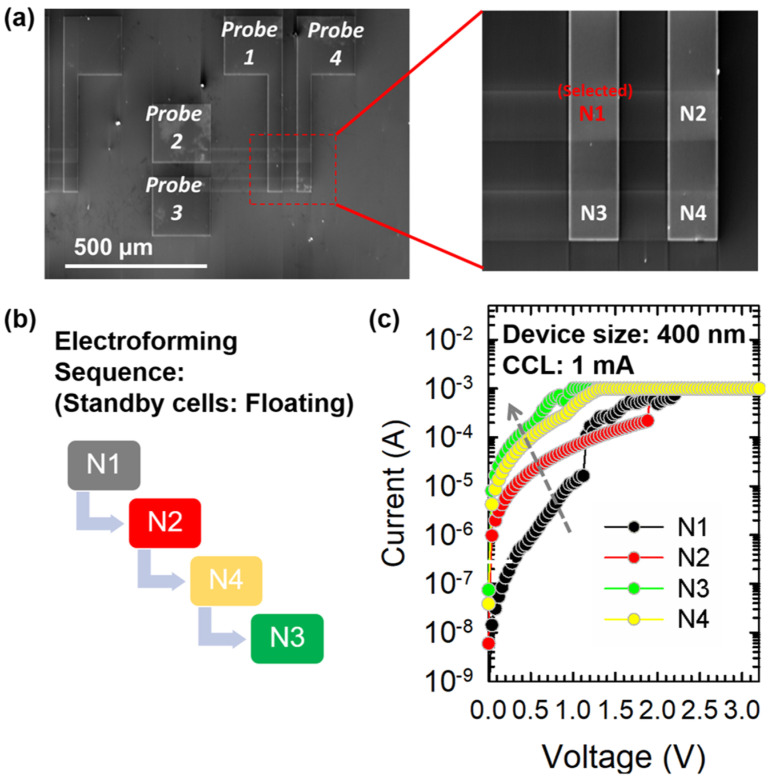
(**a**) SEM image (top view) of the 2 × 2 crossbar array and voltage is applied on the N1 cell, (**b**) electroforming sequence of N1, N2, N4, N3, (**c**) I-V characteristics of HfO_x_ (6 nm) single-layer device in the forming sequence of N1 → N2 → N4 → N3 (size: 400 nm × 400 nm, CCL of 1 mA).

The 6 nm of HfO_x_ (H) were deposited as resistive switching layers by atomic layer deposition (ALD). The HfO_x_ of 6 nm and SiO_x_ (S) of 3 nm are deposited by thermal ALD at 250 degrees C and sputtering, respectively. Titanium Nitride/Titanium was deposited as top electrodes followed by patterning. The HfO_x_ of 6 nm single layer devices are here used as a reference. Noted the device fabrication process in this work is also feasible to the embedded RRAM in complementary-metal-oxide-semiconductor (CMOS)-compatible back-end-of-line (BEOL) process [30]. The device size in the square with a side width of 760 nm is defined by the width of the tungsten plug, as showed in Figure 1c. A DC experimental setup was used to allow full electrical characterization of the select devices, and the probe numbers are associated with the cell number in the array (Figure 2a). A B1500A semiconductor parameter analyzer and Lakeshore probe station were used to measure the current-voltage (I-V) behaviors. The bias was applied to BE while TE was grounded during measurements. All devices were characterized after forming, which was carried out at positive voltage. The positive voltage is applied on the TE with grounded BE for the read and electroforming process.

## 3. Results and Discussion

The built-in SPC immunity of RRAM with intrinsic nonlinearity for suppressing the sneak path current, namely “selectorless RRAM” [31,32,33]. The nonlinearity (NL) is defined as the current at full-read voltage divided by the current at 1/2 read voltage as the V/2 read [34]. The built-in nonlinear nature can alleviate the sneak current because the on-state of the selected cell can be read at a “high-voltage” region, while the sharp conductance decrease at the “low-voltage region” effectively suppresses sneak current results from the unselected cells [35].

In this work, the 2 × 2 crossbar composed of bilayer selector less RRAM is fabricated and investigated. To initiate the resistive switching, a single sweep *electroforming* process was used, which includes a current-limited voltage sweep to induce soft breakdown to electroform the filamentary structure. In the crossbar array, the electroforming is conducted in the sequence of N1, N2, N4, and N3 (Figure 2c). Here, the soft-breakdown process is performed by sweeping the voltage until the current abruptly increases to a compliance current limit (CCL) of 1 mA to prevent permanent hard-breakdown and increases the “electroforming yield” for RRAM devices, i.e. % of the good device after electroforming. The current before forming voltage is considered in this paper as the indicator for the sneak path currents generated from the neighboring memory cells. Interestingly, the sneak path current issue can be observed in the sequential electroformation in Figure 2c. The selected cell with firstly electroformation is intentionally chosen on N1. After applying the electroforming sweep on the N1 cell (black curve), the electroforming voltage is ~1.2 V with an abrupt current increment while all the other cells are at standby voltage as the unselected cells. The initial current of the standby cell is higher than the pristine state during the sequence of forming process, which indicates the sneak path current issue affects the initial states in unselected cell i.e. N2, N4, N3 while forming is conducted on N2, N4, and N3 cell in sequence. It is worth noting that there is no RESET process followed by electroformation, and the methodology is intentionally designed to investigate the SPC phenomena during the first step of utilized the RRAM, i.e. electroformation. The forming voltage can still be observed with the abrupt current increment while forming the N1 and N2. The forming voltage of N1 is 1.2 V, and which of N2 is 2 V. The higher N2 forming voltage is thought to be suggested the parasitic resistance on the external circuit which results in the larger forming voltage. The current before forming voltage is considered in this paper as the indicator for the sneak path currents generated from the neighboring memory cells. However, due to the SPC interference on N4 and N3 after forming N1 and N2, the current of cells is too high to remain on the high resistance state (HRS). That is, the N4 and N3 (yellow and green curve in Figure 2c) are “passively” partially electroformed due to the sneak path current contributed by electroforming N1 and N2.

**Figure 3:**
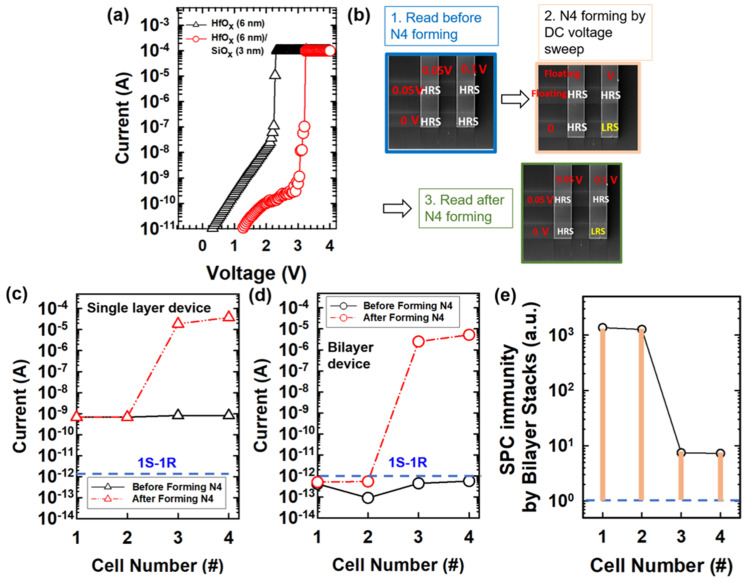
(**a**) Electroforming of HfO_x_ (6 nm)(black curve) and HfO_x_ (6 nm)/SiO_x_ (3 nm) (red curve) devices i.e. N4 cell, (**b**) the design flow of experiments,(**c**,**d**) read current of HfO_x_ (6 nm) on standby cells before (black curve) and after N4 electroformation (red curve), (**e)** SPC immunity improvement by using bilayer stacked structure.

Figure 3a shows the comparison of single-layer and bilayer stacked devices during the electroformation process. The initial current on bilayer structure is ~0.01 of which on the single-layer device at a read voltage of 2 V. The inherent SPC immunity is showed in the bilayer structure, and the SPC interference is observed on 4 cells in the crossbar array (including selected cell i.e. N4, and unselected cells i.e. N1, N2, N3). To decouple the read interference and write interference, the memory devices are programmed and read separately while comparing the varied device structures. Figure 3a show the SPC interference by N4 electroforming on HfO_x_ single layer device array and HfO_x_/SiO_x_ bilayer device array, respectively. The reading was conducted on all four memory cells before N4 forming with the half-read scheme (V/2 read scheme), as followed by the DC voltage- sweep N4 electroforming process with floating the rest of two electrodes. Then, the reading process was conducted again on all the four memory cells with the half-read scheme (Figure 3b). The black curve shows the read currents of all the cells before N4 electroformation, and the red curve shows the currents of all cells after N4 electroformation. The current after N4 electroformed is elevated in not only N4 but all the other unselected cells, especially on N3. It depicts the sneak path current generated during the reading process after N4 forming, where the N3 encountered severe SPC interferences and the current increases without electroformation on N3. Noted the reading scheme is applied as showed in Figure 3b. To investigate carefully the interferential currents, the normalized current is calculated for all the cells in the array composed of single-layer and bilayer devices (Figure 3d). The normalized current of bilayer crossbar array is 7.5–22× lowered than which of single layer crossbar array, which interprets the SPC immunity on bilayer devices was leveraged to reduce the misreading or crosstalks in the crossbar array applications. Figure 3e shows the SPC immunity is improved by ~10^3^ for N1 and N2 cells, and ~10x for N3 and N4 cell, where the SPC immunity is defined as read current of the single-layer device divided by bilayer devices. Noted the sneak path current is still affecting the bilayer devices especially on N3 and N4 due to the read interference current occurs while no cells are written at the moment. 

**Figure 4:**
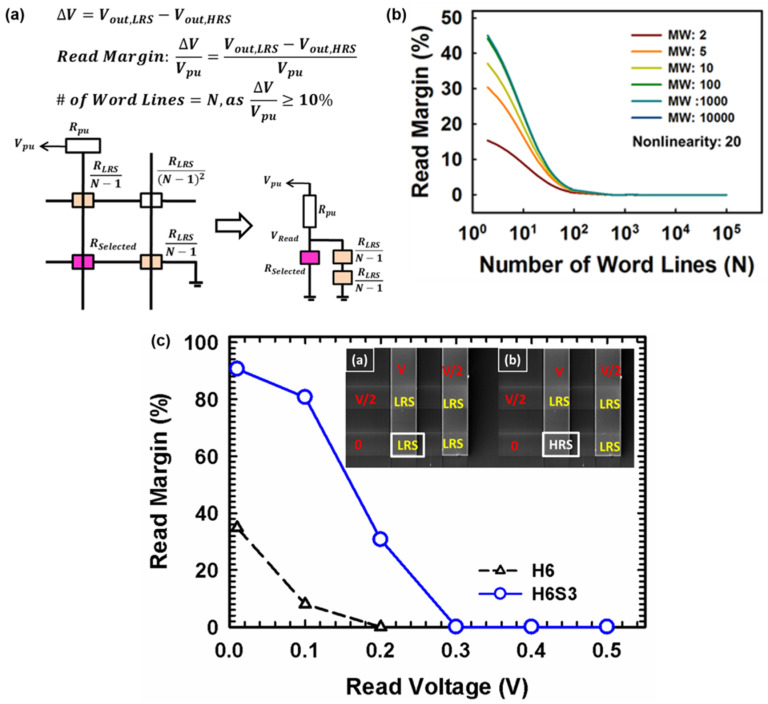
(**a**) Normalized current of cell numbers after N4 electroformation, (**b**) Simulated results with fixed NL (~20) with various memory window (~2–10^4^). The read margin remains as the MW >10^2^, (**c**) read margin with the V/2 read scheme applied on 2 × 2 array configuration with the HfO_x_ (6 nm)/SiO_x_ (3 nm) memory with self-rectifying effect, and HfO_x_ (6 nm) single-layer device.

The equivalent circuit of an N × N crossbar array for the one-bit line pull-up read scheme (Figure 4a). The read margin is defined as the voltage drop (ΔV) divided by the pull-up voltage applied externally, where the sneak path current caused the voltage drop when the selected cell is at a low resistance state (LRS) i.e less resistive and higher SPC. The selected cell (pink marked) is under the full-read voltage (Vread) and the unselected cell (orange marked) is under half of the V_read_. The pull-up resistor is utilized as the passive component for pull-up voltage (V_pu_) measurement and validation. The N represents the number fo wordline i.e. array size, and the equivalent circuit is showed that the selected and unselected RRAM cell is in parallel [36,37,38]. Herein, the second-order of neighboring cells i.e. (N-2) cell in the schematic, is neglected due to the ignorable interference current contribution. That is, the component in the read margin calculation is suggested to be neglected due to ignorable interference current contribution by second-order resistor. The equivalent circuit is simplified and includes first-order resistors in the form of (R_LRS_/(N-1)) [38].

Noted that the SPC is lowered when the selected cell is at a high resistance state (HRS) due to larger resistances in the crossbar array. The number of wordline (N) (i.e. array size) is extracted on the premise of a 10% read margin. The read margin is suggested as dominated by the SPC immunity of selectorless RRAM, and independent on memory window (MW) once MW > 10^2^ (Figure 4b). The memory window is defined as the resistance ratio of high resistive state and low resistive state i.e. on-off ratio of the memory. The read margin increases with the number of wordline decreasing, which is owing to a lower sneak path current through the unselected neighboring cells. In other words, the read margin deteriorates as memory size becomes larger and larger, where the sum of SPC is larger in a larger memory array. Besides, the device size dependency is studied with measured values of MW and SPC immunity with various device structures. The SPC immunity is independent of the device sizes for various structures, which suggests the localized filament switching and the potential for high scalability on this bilayer selectorless RRAMs (data not showed). The V/2 read scheme is applied on a 2 × 2 array, which is configured by the HfO_x_ (6 nm)/SiO_x_ (3 nm) memory cells with self-rectifying effect as HfO_x_ (6 nm) single-layer cells. 

The read margin degrades with increasing read voltages, which depicts that the sneak path currents issue in the array is worse when the read voltages increased (Figure 4c). The pull-up resistor is used for the voltage drop measurement to calculate the read margin by definition [38]. The read margin is defined as the voltage drop (ΔV) divided by the pull-up voltage applied externally, where the sneak path current caused the voltage drop when the selected cell is at a low resistance state (LRS) i.e less resistive and higher SPC. Noted the resistance of the pull-up resistor is chosen as the LRS on the cell (~5 MΩ). The read margin decreases with increasing read voltage is due to the increment of voltage drop when the cell in HRS (i.e. V_HRS_). In other words, the sneak path current generated during the reading leads to higher V_HRS_, and lower read margin. The SPC immunity and the number of possible word lines in the array are higher in high-k/low-k stacked structures (i.e. H6S3) than a single high-k layer (i.e. H6). The self-rectifying effect of the bilayer device mitigates the read margin degradation owing to the built-in nonlinear nature which suppresses the sneak path currents. The mechanism was investigated on built-in nonlinearity in bilayer selectorless RRAM (patterned single device) comprehensively, including electrical modulations, switching gap of filamentary structure, and current transport mechanism by numerical analysis [39]. The dielectric non-uniformity and switching gap location in bilayer structures need to be well-designed to implement the built-in nonlinearity and perform the SPC immunity on the RRAM selectorless crossbar array. Leveraging the dielectric behaviors on selectorless bilayer RRAM, the reduced read currently on unselected cell (~0.01× of conventional RRAM) which is utilized as the “sneak path current block” in the future high-density array applications [39,40].

**Figure 5:**
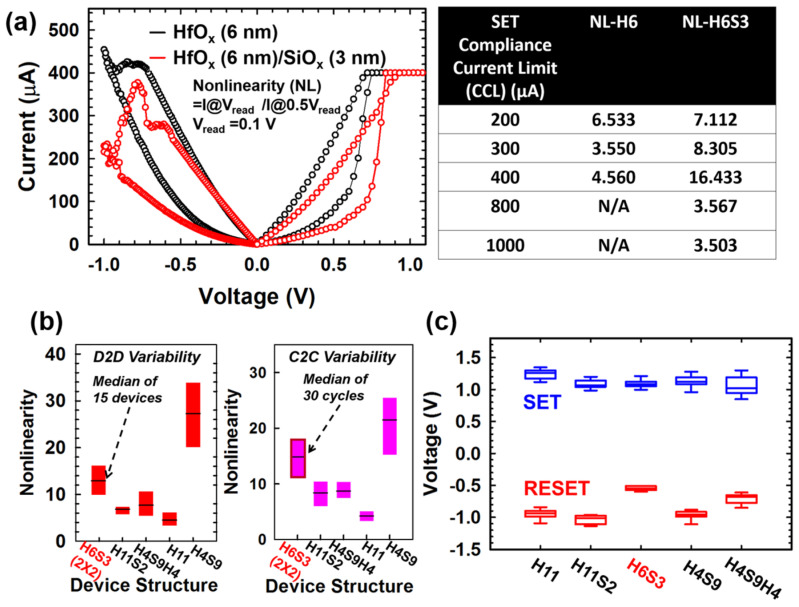
(**a**) I-V characteristics of single-layer and bilayer devices at CCL of 400µA (left), and the summarized table of intrinsic nonlinearity under SET CCL modulations, (**b**) Nonlinearity characteristics and device-to-device, cycle-to-cycle variability for various stacked structures, (**c**) switching voltages of various stacked structures.

The I-V characteristics of HfO_x_ single layer and HfO_x_/SiO_x_ bilayer devices are showed in Figure 5 (black and red curve, the median of 30 switching cycles). The nonlinearity is defined as the current at reading voltage by a current at half of the read voltage. The SET compliance current limit (CCL) is applied to investigate the intrinsic nonlinearity of bilayer and single-layer devices. SPC immunity increases with increasing nonlinearity. The optimized nonlinearity of the bilayer device (NL~20) is obtained by applying SET CCL of 400 µA. The optimization of nonlinearity with SET CCL modulation is thought to be suggested as the resistive switching gap location. The nonuniformity of bilayer stacks i.e. HfO_x_ of 6 nm and SiO_x_ of 3 nm can be leveraged for the optimized NL with specific current control, according to the filamentary structures formed in the bilayer dielectric stacks [28]. To understand the NL behaviors of bilayer stacked devices, the device-to-device (D2D) and cycle-to-cycle (C2C) variability has been investigated in Figure 5c. There are 15 devices for each structure and 30 cycles for each device have be measured. It is worth to noted the considerable improvement of nonlinearity (~16, cycle-to-cycle) in HfO_x_ (6 nm)/SiO_x_ (3 nm) in the 2 × 2 array has been observed as compared to the single layer HfO_x_ (11 nm) as the individual device without interferences from the neighboring cells. The statistical results of the variability in cycling test has also been studied, which is less than 1 order of magnitude in HfO_x_ (6 nm)/SiO_x_ (3 nm), and not deteriorate the SPC immunity. In contrast, the variability is larger than 1 order in HfO_x_ (4 nm)/SiO_x_ (9 nm), which is suggested to have the relatively larger impact on SPC immunity due to the larger D2D and C2C variability. Additionally, the switching voltages of the bilayer devices for various structures (device size 400 nm × 400 nm), has been investigated (Figure 5c), The SET and RESET voltages are reduced (SET: 1 V; RESET: −0.5 V) in the HfO_x_ (6 nm)/SiO_x_ (3 nm) device than other structures, which is suggested the total oxide stacks are thinner (<10 nm) results in the lower switching voltages for filamentary switching [41].

## 4. Conclusions

In this work, the sneak path current problem is observed and investigated by the electrical experimental measurements in a 2 × 2 crossbar array structure. The sneak path current results in crosstalk in unselected cells can be mitigated by the single-layer HfO_x_ device, and the sneak path current is reduced ~20% in a bilayer structure. The voltage-read stress-induced read margin degradation has also been investigated, and the read voltage with 10% of the reading margin is elevated to 2.5 V in bilayer device with good SPC immunity as compared to 0.1 V in single layer device. The oxide-based bilayer stacked RRAM offering immunity toward sneak path currents is developed as a selectorless RRAM and utilized in high-density memory integrations while implementing the future high-density storage and in-memory computing applications.
